# Treatment of periodontal disease using xanthan based chlorhexidine gel

**DOI:** 10.6026/97320630017326

**Published:** 2021-02-28

**Authors:** Abhishek Gautam, Kumar Manish, Raju Kumar

**Affiliations:** 1 Department of Dentistry, Anugrah Narayan Magadh Medical College and Hospital, Gaya, Bihar, India; 2 Indian Agricultural Statistics Research Institute, New Delhi, India

**Keywords:** Chlosite, chronic periodontitis, xanthan - based chlorhexidine

## Abstract

People of all ages are suffering from periodontal disease. It causes indirect damage in the oral cavity. It is of interest to evaluate the efficacy of xanthan-based chlorhexidine gel (Xan-CHX) in patients with mild-severe chronic periodontitis. Five patients with 60 sites were divided in two groups. Group A (treated with SRP) and group B (treated with Chlosite i.e., SRP + CHL). The recorded clinical parameters were Plaque index (PI), Gingival index (GI), Bleeding index (BI), and Clinical attachment Level (CAL) with sub gingival plaque subjected to microbial analysis. Significant reduction was observed in both groups. However, group B (treated with Chlosite i.e., SRP + CHL) showed statistically significant improvement on above mentioned parameters as compared to group A. Data suggest that in the treatment of periodontal disease (viz. PI, GI, BI and CAL) combination of SRP and Chlosite showed added benefits over only SRP.

## Background

Tooth loss occurs due to periodontal disease which causes infection that leads to breakdown of periodontal structures in addition to damage of alveolar bone [1,2]. Scaling along with root planning has been used routinely for plaque control. It provides long-term stability to patients however; pathogenic bacteria may not eliminate completely [3]. Since 1970's delivery of antimicrobial agents for reduction and elimination (viz. tetracycline, doxycycline, metronidazole, minocycline etc.) of periodontal pocket as well as reformation of healthy dento-gingival junction have been recognized in periodontal therapy [4,5]. Excess use of antibiotics may result in risks viz. bacterial resistance, side effects, drug interactions etc [6]. To overcome of above discussed risk, discovery of Chlorhexidine product has been accomplished as useful methods. In recent years, study suggest that Xanthan based Chlorhexidine (CHLOSITE® GHIMAS) have been found very effective antimicrobial agent against supragingival plaque bacteria for prevention of gingivitis [7]. Therefore, it is of interest to evaluate the efficacy of xanthan-based chlorhexidine gel (Xan-CHX) in patients with mild-severe chronic periodontitis.

## Materials and Methods:

Periodontal pockets measuring 5 to 7 mm from different quadrants of mouth from 60 sites of five patients were selected from Outpatient Department of Dentistry, Anugrah Narayan Magadh Medical College and Hospital, Gaya, Bihar. The age group of the patients suffering from chronic generalized periodontitis was in between 25 to 55 years. The selected patients have signs of bone loss on clinical and radiographic examination and did not receive any periodontal therapy for past 6 months. However, patients with hypersensitivity, pregnant as well as lactating woman, and smokers were excluded. Each selected sites were separated into two groups (Table 1) with following clinical parameter: Plaque index (PI, Silness and Loe) [8], Gingival index (GI, Loe and Silness) [9], Bleeding index (BI, Ainamo and Bay) [10], Clinical attachment level (CAL) using UNC-15 periodontal probe.

## Clinical parameters:

1) Baseline oral examination

2) Plaque index.

3) Gingival index.

4) Probing depth and clinical attachment level measured by UNC-15 probe (Figure A)

5) Scaling and root planning performed on each tooth using ultrasonic scalar and hand instruments (Figure B)

6) Pocket irrigation with normal saline. 

7) Drying of pocket with paper points (Figure C)

8) Chlorhexidine gel delivered in specific pockets of teeth included in Group B (Figure D)

9) Applied directly from the commercially supplied syringe into the pocket. 

10) Mode of application - Special needle:

a. Blunt tip. 

b. Lateral opening. 

c. Facilitates gel application without traumatizing periodontal depth tissue. 

11) Gel injected:

a. First into deepest part of pocket. 

b. Continuing to extrude the material, the needle slowly withdrawn till it reached the superior portion of pocket.

## Recall visit:

1) 1 month after treatment.

2) Oral examination done. 

3) Periodontal examination done.

4) Measurements recorded.

Figure (A) UNC probe and periodontal hand instruments, (B) Ultrasonic scaler, (C) Paper points in place to dry the periodontal pocket, (D) Delivery of Chlorhexidine gel

## Results:

Calculation of the mean of clinical parameters at base line as well as one month recall was prepared by Student's paired t-test (SPSS software, version11.5). The p value<0.001 was considered highly significant. Baseline visit mean plaque score was 1.70±0.35, which decreased to 0.86±1.10 after 1 month in Group A (p <0.001), whereas it was 1.72±0.56 at baseline visit and 0.52±1.10 after 1 month in Group B (p<0.001). Baseline visit mean gingival index in Group A was 1.58±0.48 and 0.76±0.18 after 1 month (p<0.001). Group B's baseline visit mean gingival index was 1.64±0.16, which became 0.46+0.34 after 1 month (p<0.001). Baseline visit mean pocket depth was 6.45±+0.98 in Group A and 5.60±1.30 at 1month (p<0.001). Group B's mean pocket depth was 6.52±0.92 and 4.76±1.18 at baseline visit and 1month respectively (p<0.001). Mean clinical attachment level was 6.95+1.20 in Group A at baseline visit and was 6.00±1.40 after 1month whereas for Group B it was 6.86±1.10 at baseline visit and 5.12±1.16 after 1month (p<0.001) (Table 1).

## Discussion:

Chronic periodontitis have been found common in the people aged between 30 - 50 years. Oral health can be controlled by control of plaque in the treatment of periodontal disease11. Although removal of difficulties in the maintenance of oral hygiene due to plague, the most efficient anti-plaque agent is chlorhexidine which is found suitable these days. The result of the present study have shown that use of chlorhexidine (xanthan-based chlorhexidine gel and 0.2% chlorhexidine irrigation) followed by scaling and root planning (SRP) reflects a significantly better and greater improvement in clinical parameters when compared with the improvement obtained with SRP alone without any adverse effects reported by the patients during study. Gingival and plaque indices remain satisfactory due to proper oral hygiene maintenance and thoroughness of SRP that showed significant reduction in PI & GI at 30 days follow up visit when compared to baseline levels in all groups but marked reduction was seen in group B when compared to group A. Apart from PI & GI Group B showed a slightly greater gain in the CAL when compared to Group A. This positive improvement in CAL achieved due to absence of challenge during the critical initial phase of healing following SRP. Oosterwaal et al., investigated the effects of a 2% CHX gel used as an adjunct to SRP; similar clinical results were obtained with SRP treatment alone and when subgingival administration of 2% CHX or placebo gels were associated with SRP.12 Quirynen et al., reported negligible beneficial effects over SRP alone when a 1% CHX gel was subgingivally administered as an adjunct to SRP in a one-stage full mouth disinfection protocol.13 Unsall et al., found less CAL gain in periodontal sites treated with SRP and subgingival administration of 1% CHX gel compared to those treated with SRP alone.14 Xanthan gum has been shown to have bioadhesive properties and provided the most prolonged adhesion time on the oral mucosa with respect to other delivery vehicles.15

## Conclusion:

The application of Xan-CHX gel in the treatment of chronic periodontitis is reported. Further studies are needed with a larger sample size in this context.

## Figures and Tables

**Table 1:** Inter group comparison of clinical parameters

Parameters	Group-A			Group-B		
	Baseline	1 months		Baseline	1 months	
	Mean±SD	Mean±SD	P value	Mean±SD	Mean±SD	P value
Plaque index	1.70±35	0.86±1.10	<0.001	1.72±0.56	0.52±0.09	<0.001
Gingival index	1.58±0.48	0.76±0.18	<0.001	1.64±0.16	0.46±0.34	<0.001
Pocket depth	6.45±0.98	5.60±1.30	<0.001	6.52±0.92	4.76±1.18	<0.001
Clinical attachment level	6.95±1.20	6.00±1.40	<0.001	6.86±1.10	5.12±1.16	<0.001
